# A New Dawn in Nationalism Studies? Some Fresh Incentives to Overcome Historiographical Nationalism

**DOI:** 10.1177/0265691417741830

**Published:** 2018-01-11

**Authors:** Eric Storm

**Affiliations:** Leiden University, the Netherlands

**Keywords:** Banal nationalism, global perspectives, methodological nationalism, nationalism, nation-building

## Abstract

Nationalism studies does not seem to be a very innovative field of research. The path-breaking views of Anderson, Gellner and Hobsbawm – all published in 1983 – still form the starting point for almost all existing investigations. Moreover, most recent studies focus on one national case, which implicitly results in a vast collection of ‘unique’ trajectories. However, over the last few years a number of highly original studies on the origins of nationalism, nation-state formation, banal nationalism, methodological nationalism and nation-building in a global perspective seem to announce a new dawn. Some of these refreshing interpretations – which will be discussed in this article – clearly demonstrate that historiographical nationalism still has a preponderant role in history writing. In the concluding paragraphs I will emphasize the need to overcome not only methodological nationalism, but also the terminological and normative nationalism that still dominates our discipline.

It seems that nothing really new has happened with nationalism studies over the last three decades. The path-breaking works that were published in 1983 – Benedict Anderson’s *Imagined Communities*, Ernest Gellner’s *Nations and Nationalism* and Eric Hobsbawm and Terence Ranger’s *The Invention of Tradition* – still provide the interpretative framework for almost all existing studies. Moreover, over the last three decades an enormous amount of case studies have been published that focus on one national context, so it is difficult to get an overview of the entire field. As a consequence, the differences between the various national cases have received more attention than the similarities. Recently, however, a few books have appeared on the market that not only seriously update the now classic views of these authors, but can potentially sit alongside them in the pantheon of nationalism studies. Moreover, they could help us overcome the historiographical nationalism that still largely dominates our discipline.

## Origins

One of the most hotly contested issues related to nationalism concerns its origins. Most experts subscribe to the modernist view which holds that nations, nation-states and nationalism are closely related to the rise of modernity and are not possible before the eighteenth century. The modernist consensus is recapitulated in *The Oxford Handbook of the History of Nationalism*, a recent global overview edited by John Breuilly.^1^ It starts, unsurprisingly, with a chapter on the intellectual origins of nationalism focusing on Rousseau’s plea for popular sovereignty and Herder’s idea that each nation had its own unique culture, while the first territorial chapters deal with the Wars of Independence in the Americas and the impact of the French Revolution in Europe. By clustering various neighbouring countries in regional chapters, it avoids the traditional inward-looking case-study approach that generally focuses only on those nationalisms that resulted in independent nation-states. Some authors make use of this comparative approach to test more general theoretical insights. Thus in his chapter on South-East Asia, David Henley thoroughly examines why French Indochina was divided into three independent nation-states, while the Dutch East Indies – which was probably much more diverse – became a single Indonesian nation-state. According to him, the memory of the ancient kingdoms that formed the basis for the new states was still alive in Indochina, whereas major pre-colonial states had disappeared centuries ago in the Dutch East Indies. The Indonesian nationalists therefore took over the colonial state instead of creating a national state on the ethnically homogenous central island of Java. Breuilly himself approaches his chapter on national unifications in nineteenth-century Europe in an original way by also including the Polish case. He thus sheds new light on the well-known process of Italian and German nation-state formation. He even pleads for substituting the finalistic term ‘unification nationalism’ for ‘pan nationalism’, which aims at uniting all members of a supposed nation in one state, but which is not necessarily successful.

The modernist view has been criticized by specialists of early modern history, who argue that many national identities are already discernible before the late eighteenth century, while authors such as Anthony Smith have emphasized the continuity between national and older ethnic identities.^2^ A forceful attack on the modernist interpretation has been launched recently by the political scientist Azar Gat in an ambitious book on the long history and deep roots of political ethnicity and nationalism, simply titled *Nations*.^3^ Inspired by sociobiological theories, he argues that human beings have an innate propensity to prefer people of a closer kin to ‘strangers’. As a consequence, even in the distant past people identified with members of their own tribe, ethnicity or nation and, if needed, collaborated with them to expel ‘foreign’ invaders or alien overlords. Since he is a specialist of military history and has written a similar sweeping overview on warfare, Gat probably puts too much weight on the rather exceptional state of collective defence against an external enemy, which almost regardless of the period he interprets as a proof of nationalism or at least political ethnicity.^4^ He even argues that Ancient Egypt already was a nation-state and although he acknowledges the massive transformation that took place with the arrival of modernity, he clearly emphasizes the continuity between pre-modern and modern forms of nationalism.

Even though Gat’s views have been widely criticized, his book contains a number of fascinating critiques of the mainstream modernist interpretation.^5^ Thus, contrary to Anderson, for instance, he claims that the creation of imagined communities was not only made possible by a process of secularization and the rise of print capitalism, but could also be produced by the spoken word – for instance from the pulpit – and via religious rituals. Moreover, religions often propagated the national idea and churches were generally organized nationally; during the Reformation era this was obvious for both the Protestant and Catholic Church, but it also applies to the Orthodox parts of Europe. He further claims that Gellner and other modernists have focused too much on France as the model of a successful nation-state and on the Habsburg Empire or India as typical multi-national dynastic empires without a dominant ethnic group. He argues, however, that Spain and England were much more representative nation-states and unlike France they did not totally succeed in assimilating the ethnically different periphery: Portugal, the Basque Country and Catalonia in the case of Spain and Ireland, Scotland and Wales in Great Britain. Moreover, large Eurasian empires with a strong ethnic core such as Russia, China and Japan were more common according to the author than cases such as India or the Habsburg Empire. Although, with his taste for polemics, Gat probably overstates his argument, historians recently have tried to show in theoretically well-informed case studies that collective (national) identities already played a role in the early modern period.^6^ Nevertheless, without popular sovereignty, national citizenship and sharp territorial boundaries, the differences with modern nationalism and the nation-state remain enormous.

The modernist interpretation of the origins of nationalism is updated in Siniša Malešević’s handbook *Nation-States and Nationalisms: Organization, Ideology and Solidarity*.^7^ The author combines Gellner’s focus on the role of industrial modernization in requiring mass education in a standard language, Anderson’s emphasis on cultural factors such as the undermining of royal authority by the growing impact of secularization and the role of the printing press in diffusing national standard languages, with more recent views on the impact of state-building. According to Malešević, the modern, bureaucratic state came into existence in Europe through a slow process that lasted several centuries. Over time, its potential to construct and enlarge the country’s infrastructure grew enormously and it began to reach deep into the lives of its inhabitants. At the same time, increased literacy, the growing role of the media and the rise of the public sphere enabled the ideological mobilization of the masses, and it was nationalism that provided a highly successful state-centred ideological doctrine that potentially encompassed all citizens. In order to make the bureaucratic nation-state attractive, fostering solidarity among the members of the nation became crucial and as a consequence it was portrayed as a close-knit community. The nation-state finally succeeded because it resonated in ‘hubs of micro-solidarity’, such as neighbourhoods and private homes. All this, according to the author, was not possible before the late eighteenth century. In another recent study, Charles Maier approaches the nationalizing impact of state-building from a slightly different angle by focusing on the construction of borders, a process that began in seventeenth-century Europe and increasingly led to the homogenization of both territory and people.^8^

In 2013, the sociologist Andreas Wimmer also published an ambitious new view on the rise of nationalism and nation-states based on both quantitative and qualitative methods. According to him, nation-states are neither an ideological construct imposed from above, nor the result of an ‘eternal desire for ethnic self-rule’, but the result of political modernization. Indirect rule by provincial elites of earlier state forms was undermined by administrative centralization and the growing clout of the masses, for instance through new forms of warfare (such as the *levée en masse*), increased political awareness or the rise of voluntary organizations. This meant that dominant elites could no longer depend on their alliances with subordinate elites, but had to collaborate with at least a part of the masses. Political modernization could have three different outcomes according to Wimmer’s model: (a) nation-states in which the central elites succeeded in integrating the entire population; (b) populist nationalism in which the dominant elite allied with the masses against a subordinate elite; and (c) ethnic closure, which means that both dominant elites and subordinate elites allied with their own ethnic constituencies. As a consequence, populism, large-scale ethnic conflicts and nation-states are all products of modernity. The main factor that determined successful nation-building was not ethnic homogeneity, but state centralization. In strong centralized states it was much easier for dominant elites to reach a new exchange relation with the population at large: taxes and military service in exchange for public goods and political participation.^9^

Nevertheless, in Wimmer’s view, it is important to distinguish between the rise of the first national communities – in France, the United States and Britain – during the late eighteenth century and the dissemination of the nation-state model to the rest of the world during the nineteenth and twentieth centuries. Since the new nation-states were militarily and politically more powerful, political leaders elsewhere began to adopt the model in their own territories, while intellectuals began to extol the nation-state as a more legitimate model that would bring higher rewards to the population at large. Economic, cultural or political modernization, for instance through industrialization, growing literacy rates or state centralization, are much less important for explaining the rise of nation-states elsewhere in the world than the specific power configuration at a given time and place, even though the route taken could vary from a gradual transition, revolution, secession, civil war or unification. Nationalists had little chance in strong and stable empires, while substantial power shifts, wars and the establishment of nation-states in surrounding areas provided opportunities for nation-state formation.^10^ Another surprising conclusion of Wimmer’s ground-breaking study is that, contrary to existing views, wars were more likely to occur during and after the transition to the nation-state model. His analysis is based on a sophisticated statistical analysis of new sets of data for both civil and interstate wars during the last two centuries. Although his social science approach sometimes is quite abstract and schematic, his book should be a starting point for all scholars who want to study the rise of nationalism or the relationship between nation-state formation, ethnic politics and armed conflict.

## Nationalism in Everyday Life

Another issue that has attracted sustained scholarly attention is the nation-building process. Influenced by Eugen Weber’s *Peasants into Frenchmen* and Eric Hobsbawm’s concept of ‘invention of tradition’, historians have conducted a large number of investigations on conscious nation-building activities by national and regional elites and the invention of specific nationalist traditions such as national holidays, symbols, flags, commemorations and monuments.^11^ However, social scientists have recently begun to pay attention to more mundane forms of nationalism, which often were not produced consciously by dedicated nationalist activists, and which are still largely ignored by historians.

Crucial in this sense is Michael Billig’s *Banal Nationalism*, which was published in 1995.^12^ According to Billig, nationalism expresses itself openly not only in wars, revolutions and national holidays, but also in banal or everyday experiences such as sport, travel, mass media and at home. According to Billig, the identification with one’s own nation does not only occur when nationalism is ‘hot’, but also when it has ‘cooled down’. And this happens on a daily basis in all kinds of ‘trivial’ or ‘banal’ acts, which are probably more pervasive because they pass largely unnoticed. When we speak about ‘the government’, ‘the economy’ or ‘the countryside’, we generally understand that we refer to the government, economy or countryside of our own country. The identification with ‘our’ soldiers, sportsmen and singers is taken for granted as a natural phenomenon, as is the division between foreign and domestic news. In the forecast even the weather seems to abide by national frontiers. In fact, the nation-state has become internalized as the ‘natural’ unit by which we divide the world.

Billig’s already classical study and Tim Edensor’s equally influential *National Identity, Popular Culture and Everyday Life* have produced a wave of studies on more mundane forms of nationalism.^13^ However, most of these studies are conducted by social scientists and focus on very recent periods. A good example is *Food, National Identity and Nationalism* by Atsuko Ichijo and Ronald Ranta, which shows that even such banal practices as eating and drinking have been thoroughly influenced by nationalism.^14^ Through various short case studies they analyse how individuals, groups, companies, states and international organizations routinely connect specific food items or cooking practices to specific nations. Sometimes, this is the consequence of conscious activities by nationalists. Thus, in the case of Scotland, the authors make clear that from the early nineteenth century onwards nationalists used to gather on 25 January for a Burns Supper to celebrate the national poet’s birthday. Toasts, songs and poems were combined with whisky and ‘national’ dishes such as haggis. Probably more influential in connecting specific beverages with the nation were the activities of the Scotch Whisky Association, which – following the example of the French wine growers – tried to defend its economic interests by defining whisky as indissolubly connected to the soil and traditions of Scotland.

Today, however, the nationalization of food is primarily driven by commercial companies, such as those in the (British) catering industry. In order to demonstrate the ‘authenticity’ of the almost industrially prepared dishes they are presented with a specific nationality, such as Moroccan tagine, French onion soup or Greek-style lamb. This also helps to diversify the menu. Moreover, in order to increase the transparency of the production chain and in many cases also to stimulate sustainability, coffee, tea, chocolate, but also meat, vegetables, fruit and dairy products are labelled as originating from and belonging to a certain nation or one of its regions. Probably the most paradoxical outcome of the book is that international organizations that promote universal values, such as UNESCO, indirectly help to reify national identities. Thus, the intangible heritage programme that started in 2003 is explicitly meant to protect the world’s cultural diversity. However, since applications can only be made by member states and the immaterial traditions that are proposed for inscription in the official list should not be confined to certain small groups or elites, most gastronomic traditions that have been recognized so far are national traditions, such as the ‘gastronomic meal’ of the French, Turkish coffee culture and traditional Mexican cuisine. Thus, UNESCO’s immaterial heritage programme ends up subsuming the local under the national, while supporting national claims to culinary ownership.

Most social scientists associate banal nationalism with stable, post Second World War nation-states. Nevertheless, in *Nation-States and Nationalisms* Malešević argues that trivial forms of nationalism have played an important role since its rise in the late eighteenth century. He thus mentions the dress of French revolutionaries, the Englishness in early nineteenth-century novels and the role of world fairs in disseminating stereotypical images, products and experiences of participating nations.^15^ Historians have not yet paid much attention to these kinds of topics, although there are a few fascinating exceptions. By focusing on the nationalist motivations of commercial actors, authors such as Guy, Harp and Zuelow have shown how champagne became an essentially French beverage, how a company like Michelin tried to sell more tyres through all kinds of nationalist activities and how tourism stimulated Ireland to become more Irish.^16^ However, the unintended nationalizing consequences of modernization and standardization processes, or the banalization of various nationalist symbols and practices are more difficult to study for historians. Detailed information on the conscious invention of national traditions is often easily discovered in archives and the contemporary press. However, it is much more difficult to find primary sources that could shed light on how certain traditions over time became widely accepted, especially since for the more distant past we cannot make use of interviews or opinion polls. Creative solutions are definitely needed. Perhaps humorous texts and pictures could be an entry into detecting what has only quite recently become accepted as standard.

## Methodological Nationalism

Not only has the impact of nationalism on daily life been much more pervasive than could be inferred from older studies, but nationalism has also heavily influenced the way scholars have studied human society. Thus, in an article published in 2002, Andreas Wimmer and Nina Glick Schiller had already made clear that the social sciences take the nation-state for granted and that research is affected in three different ways by ‘methodological nationalism’. First, classical social theory downplays the role of nationalism and sees it as something backward, but at the same time accepts a world divided into nation-states as a given. Second, sociologists equate society with national society and thus take nation-states as the ‘naturally given entities of study’. Third, in the social sciences the analytical focus is reduced to the boundaries of the nation-state and everything that extended ‘over its borders was cut off analytically’. The consequence of this use of the nation-state as a container in which developments are analysed is that statistics are routinely gathered for national units, trans-border movements or influences are ignored and immigration, for instance, is seen as an anomaly that should be controlled and monitored, while this is not the case for migration flows within the borders of a nation-state.^17^

Many of these critiques also apply to the discipline of history and in fact one of the main conclusions of the European Science Foundation research programme ‘Representations of the Past: The Writing of National Histories in Nineteenth and Twentieth Century Europe’ is that history writing is largely dominated by ‘historiographical nationalism’. In the final summary of this project, *The Past as History: National Identity and Historical Consciousness in Modern Europe*, Stefan Berger argues that since the early nineteenth century historians have tended to investigate, narrate and interpret the past through a national lens.^18^ Without dealing extensively with the debate on the supposed modernity of nations and nationalism, Berger shows how from the late Middle Ages onwards references to peoples or nations became increasingly common in the work of chroniclers and historians. Nevertheless, nations were defined in a rather vague way, while historians showed more interest in the role of dynasties or the traces of God’s presence in the past. In the early modern period humanists and enlightened scholars in Western Europe began to produce more secular interpretations in which nations and even patriotism began to play a role. However, until the eighteenth century, the histories of cities, dynasties or civilizations were more important than the supposedly unique trajectory of the different nations.

In the German lands during the Romantic era the discipline of history was professionalized and institutionalized through the creation of the first university chairs. The new scientific standards of professional history writing quickly became the model for the rest of the continent, while conveying an ‘aura of truthfulness’ and ‘scientificity’. Transnational networks of scholars and international conferences played a stimulating role in transferring all kinds of innovations. However, as a reaction to the enlightened universalism and its forceful promotion by the French Revolution and Napoleon, German ‘historicism’ clearly prioritized nations over other territorial units. National history thus became the dominant form of history writing. Everywhere ‘national master narratives’ were constructed, which in many ways still dominate present day historiography. In those areas where nations already had their own state these narratives focused very much on the state, its primary decision-makers (princes and politicians) and its borderlands. A focus on the ‘people’ was more important in the case of ‘nations’ that did not yet have a state. In both cases, there was a lot of attention focused on the origins of the nation in the mists of time, on the Middle Ages as the crucial period for nation formation, on the nation’s supposed Golden Age and on the construction of canons of national heroes (and villains). Regional histories became building blocks of the national story, while religion and class were increasingly studied within a national framework.

Thus, historians interpreted the European Wars of Religion not only from an explicit national perspective, but also from a specific understanding of the collective personality of the nation. Even eminent historians did not focus on the conflict as a whole, but on those episodes that took place on the territory of one later nation-state, its impact on the history of this specific nation and how it was shaped by and impacted upon the national character that according to these historians was already in place. Karl Lamprecht, for example, argued that Protestantism contributed to the arrival of a new age of individualism in Germany.^19^

The first half of the twentieth century can, according to Berger, be characterized as the high point of historiographical nationalism. Borderlands were increasingly contested by historians, who forcefully defended the rights of their own nation. Some scholars even justified ethnic cleansing and genocide. Only in the 1960s and 70s did new historical trends, such as cultural history and history from below, begin to move away from the nation as the dominant frame of analysis. Although a more critical attitude towards national master narratives nowadays seems to have become common property among professional historians in most parts of Europe, this is not the case with public history. Thus recent handbooks directed at a broad audience, such as Heinrich August Winkler’s *Germany: The Long Road West* and Norman Davies’s *The Isles: A History*, still present a rather uncritical national narrative.^20^ Moreover, other recent approaches, such as comparative history and memory studies, generally still take the nation-state as their basic unit of analysis. Berger’s admirable tour de force really makes the reader aware of the profound and lasting impact of historiographical nationalism and the urgency to rethink the foundations of our discipline.

## Some Innovative Interpretations

The implicit consequences and inconsistencies of the current hegemony of the nation-state model are discussed in Rogers Brubaker’s *Grounds for Difference*, in which he reflects on the interconnections between social differences and collective identities. In most essays, the author first shows the inconsistencies of a specific influential current-day theory and then comes in with his own perspicacious observations and counter-arguments. Thus, in the last chapter he takes on Shmuel Eistenstadt’s theory of multiple modernities. Eisenstadt asserts that currently there are a number of cultural and political models that do not conform to the Western standards, and which even tend to become more prominent. By assuming the existence of multiple modernities, he aims to substitute older modernization theories that presupposed that the world would converge around a single, Western model of modernity. Brubaker, however, argues that in order to define societies, models or ideas as modern – even if there are multiple variants – one needs to have a clear criterion to define what all forms of modernity share. So paradoxically one again ends with a single modernity. But Brubaker also disagrees from a more historical or sociological perspective by focusing on nation-states and nationalism as central elements in the interpretation of modernity. According to him, the organizational models and ideas about ‘nation, state, citizenship and popular sovereignty form a kind of package’, and it was this package as a whole that was diffused globally, starting with the French Revolution in 1789.^21^ This does not mean that we have to adopt an outdated, Eurocentric understanding of the modern state and of nationalism as purely secular and civic. The nationalist package has proven to be flexible and adaptable enough to be adopted in many different circumstances.

In a similar way, Brubaker refutes Charles Tilly’s analysis of social inequality in *Durable Inequality*. Whereas Tilly argues that exploitation and social closure – the exclusion of outsiders from a specific profession or resource – often occurred along ethnic or gender lines, Brubaker makes clear that they do not depend on categorical differences. Moreover, by taking the nation-state as his unit of analysis, Tilly and many other social theorists overlook the main instrument of social closure in the current meritocratic world: citizenship. According to Brubaker, citizenship ‘is an *inherited* privilege (or disability), and one that is transmitted, in turn, to one’s descendants’.^22^ It is obvious that social closure based on citizenship in the modern state system did not cause the vast inequalities between the various countries, but it serves to perpetuate them. It determines one’s access to education, health care and other benefits and one’s right to work and to secure residence.

In another chapter Brubaker deals with the implications of the rise of the nation-state model during the last two centuries. This model presupposes that the borders of the state overlap with those of the nation and that a national culture is dominant within the territory of the state, but stops at its borders. Moreover, all residents should be citizens and participate in the same national culture. Although probably not a single state totally conforms to this ideal type, it constitutes the norm and as such has enormous consequences. The nation-state became a fluid space of internal geographic and social mobility. This is seen as normal and should be facilitated, while migration across national borders is considered an anomaly that undermines ‘the cultural homogeneity within states’ and the ‘sharp cultural boundaries between them’.^23^ The same is true for the existence of minority groups who formally or informally are not really part of the nation. This generally results in politics of belonging, which can have an enormous impact on those who should be included or excluded, and can even affect those fellow nationals who happen to live outside of the border. Should a migrant first become a member of the culturally defined nation before he or she can acquire citizenship? And, to provide a concrete case, do ethnic Germans from Russia have a greater right to obtain German citizenship than children of Turkish migrants who have been born and raised in Germany?

Equally lucid is the essay on the ways in which language and religion are intertwined with ethnicity and nationhood. Brubaker asserts that in premodern societies language was a private matter that was of no concern to the state, whereas the authorities often imposed religious uniformity. In present-day liberal societies, in turn, religion is a private issue, whereas the state imposes one specific language (or sometimes allows several) by making it the official medium of communication which is used in education and by institutions. Moreover, by taking the nation-state model as the norm, states generally only award official status to those minority languages that are long established within the country and not to the languages of immigrant communities. The use of a standard language in the public sphere has become so pervasive that immigrant communities rapidly adopt the dominant language of their place of residence, while the reproduction of minority languages requires sustained efforts by the state, or the limitation of the language options for specific parts of the country, as has happened in Belgium, Switzerland and Spain. This means that languages as Herder’s ‘carriers of distinctive world views’ have lost much of their ‘thickness’ (if that has ever existed at all), since over time all inhabitants – regardless of their cultural background – are socialized within the same national language. Religion, on the other hand, is routinely passed on from one generation to the next, while in most states various religious denominations have become institutionalized. As a consequence, language has lost most of its mobilizing potential, while religion, with its comprehensive set of norms, increasingly affects the public realm. Brubaker thus sees a reversal of a long-term trend, as religion today is more important than language for the ‘political accommodation of cultural difference in Western liberal democracies’.^24^

*Nationalizing Empires*, edited by Stefan Berger and Alexei Miller, aims to redefine the relationship between empires and nation-states, while overcoming the strict divide between colonial history and the study of the West.^25^ Empires and nation-states have traditionally been seen as opposites by historians. As proof of the success of Woodrow Wilson’s collating of democracy and national self-determination, empires were seen as autocratic, feudal and anachronistic. The Habsburg, Romanov and Ottoman Empires were ‘prisons of nations’, and their collapse at the end of the First World War was interpreted as the inevitable outcome of a long process of decline.^26^ Berger and Miller, however, argue that during the long nineteenth century most nation-states actually were empires. This was the case with Great Britain, France, Spain, Portugal, the Netherlands, Belgium and Denmark. Moreover, new nation-states such as Germany and Italy rapidly began their own colonial project. In order to be respected, a nation-state needed to have an (overseas) empire. On the other hand, nationalism also strongly affected the cores of the continental empires in Eastern Europe. Berger and Miller thus rightly conclude that most nations emerged within empires, that nation-building generally happened within an imperial context and that nation-building and empire were closely entangled.^27^

The impact of nationalism in the core of ten of these empires is analysed in individual chapters. Some authors focus more on one aspect – economics and migration in the case of Austria-Hungary, intellectual history in the case of Venice/Italy – than others. As the timeframes of these chapters also do not exactly match, it is difficult to compare the trajectories of each of these empires. However, by adding five more thematic and comparative chapters by foremost specialists on imperial history, the final result is a very rich and dense patchwork that explores new ground.

Almost all the chapters provide fascinating new insights. Thus, Robert Aldrich makes clear how direct links between, for instance, Brittany, Corsica, Lyon and Marseille and specific parts of the French colonial empire stimulated the integration of these regions and cities into the French nation. Xosé-Manuel Núñez Seixas shows how the loss of the last remnants of Spain’s colonial empire had a contrary effect in Spain, providing a direct stimulus for Catalan and Basque calls for autonomy. Stefan Berger argues that the racialization of national discourse in the German Empire was related to both the treatment of the Polish and Jewish minorities at home and that of colonial subjects and mixed offspring overseas. Alexei Miller emphasizes that in the Russian Empire there was not much overlap between the traditional nation-building policies of the Tsar, focusing on religion and dynastic loyalty, and those of more liberal elites who were in favour of political participation. In a similar vein, the Ottoman wavering between Ottomanism, Islamism and Turkism, according to Howard Eisenstat, was the result of a ‘messy process of experimentation’ rather than a conscious choice for a specific ideology.

The most surprising contributions deal with the Napoleonic Empire and Denmark. Michael Broers discusses Napoleon’s short-lived empire, showing that French officers and officials mainly distinguished between civilization and barbarity. The former was associated with those parts of the Empire that accepted the Code Napoleon, such as the North of France, the Low Countries, the German Rhineland and the North of Italy, while the territories and social groups that persisted in ‘barbarous’ behaviour by rejecting the new regime, and which even included the West and South of France, were considered subjugated lands where a civilizing mission was badly needed. National identities, defined by language and culture, did not yet play a major role.

Uffe Østergård, in turn, shows how the composite Danish state – which in many ways was comparable to the ‘multi-ethnic’ Habsburg Empire – was dismembered, not because of the strength of nationalist agitation, but as a consequence of lost wars (thus implicitly confirming Wimmer’s views). Since Frederick VI remained faithful to his alliance with Napoleon until the very end, he had to cede Norway to Sweden in 1814. As a consequence, the dominance of the Nordic part of the population in the Empire of the Oldenbourg dynasty became weaker, but even until the 1830s German liberals from Schleswig-Holstein were united with their Danish counterparts in their opposition to royal absolutism. Only in 1848 did nationalist tensions between the two main remaining parts of the European core of the Empire (Denmark also had small colonial outposts in India, Africa and the Caribbean) increase. However, in 1864, it was the military intervention by Prussia and Austria that forced Denmark to relinquish its German-speaking territories.

Another volume, titled *The Shadow of Colonialism on Europe’s Modern Past* and edited by Róisin Healy and Enrico Dal Lago, approaches the relationship between nation-states, empire and colonialism by focusing not on the core, but on those who were the ‘victims’ of imperial expansion within Europe.^28^ Most chapters deal with regions such as Ireland, Schleswig, Alsace and the Polish-speaking parts of the German Empire, the *Mezzogiorno* in post-unification Italy and the Ukrainian-speaking parts of Interwar Poland, while determining to what extent the oppression they suffered can be defined as ‘colonial’. In fact, this is complicated because these regions were part of nation-states and as a consequence the inhabitants were citizens with voting rights. Nevertheless, the authors – most of whom are connected to Irish universities – clearly show how nation-building policies were experienced as enforced assimilation by many inhabitants of peripheral regions. Instead of uniting the nation, the measures taken by the central government often led to discontent, active opposition and in some cases even organized resistance, and as a consequence were largely counterproductive.

Nevertheless, using the term ‘colonialism’ instead of ‘imperialism’ becomes quite confusing, since colonialism is primarily associated with economic exploitation, political marginalization and cultural denigration in the oversees colonies of the European powers. Therefore, most authors conclude that in their case the situation does not totally fit the classical definition of colonialism. Probably the main exception was Bosnia-Herzegovina, which came close to being a full-blown colony of the Austrian Empire. In fact, this edited volume takes the nation-state as standard, even in a normative sense. Consequently, the authors criticize those instances in which the nation-state ideals were violated on the ground. This also becomes clear in the normatively charged vocabulary that is used in speaking of ‘imposition’, ‘exploitation’, ‘subjugation’, ‘shadow’ and ‘darkness’. Thus, contrary to *Nationalizing Empires*, this volume does not provide an alternative to the hegemony of the nation-state in the study of nineteenth- and twentieth-century Europe, but implicitly confirms it.

## Consequences and Implications

The books that have been discussed so far not only show us new approaches or underexplored territories for the study of nationalism, nation-building and nation-state formation, they should also stimulate us to fundamentally revise our ways of writing history. First of all, there is still ample work to do for historians interested in these topics. Although the debate on the supposed modern origins of nationalism remains heated, dispassionate investigations such as that of Stefan Berger on history writing or Michael Broers on the Napoleonic era can shed more light on the transformation in the meanings of the term nation and its importance during the early modern period. It also seems justified to conclude that Wimmer’s profound investigations into the global success of the nation-state model deserve to become the starting point for anyone wishing to examine the coming into existence of a particular nation-state. Specifically, his somewhat schematic theories and models will profit from empirical case studies that can validate or fine-tune some of his conclusions. Another topic that deserves attention is the impact of nationalism on everyday life, which has not yet been thoroughly researched for the past. Social scientists have already employed new concepts and approaches, while unearthing many instances of mundane forms of nationalism in the past. However, by largely ignoring the temporal dimension, it is not clear when exactly more mundane forms of nationalism arose and how more obvious forms became banalized over time.^29^

It has also become clear that the nation-state is not the only possible state form in the modern world, but a quite specific product of historical developments. Moreover, it did not become dominant right away. Even in Europe most states were empires or aspired to become one. This slowly began to change after the First World War and Wilson’s highly influential plea in favour of the right to national self-determination, but would only become more effective after the collapse of the imperial dreams of Nazi Germany and Japan in 1945. Nonetheless, it was only in the 1960s that most Western powers began to dissolve their colonial empires, while the Soviet Empire lasted until the early 1990s. Moreover, after 1989 the autonomous nation-state was also increasingly weakened by neo-liberal policies, such as large-scale privatizations and the curtailing of the welfare state, while at the same time it was eroded from the outside by the globalization of the economy, the rapid increase of travel, tourism and migration, the growing power of transnational companies and supranational organizations such as the European Union, and by the rise of Internet. Thus, one could argue that the nation-state was only truly hegemonic during a very brief period between the 1960s and the 1980s.

Moreover, nation-states hardly conformed to the ideal type. Healy and Dal Lago’s volume shows that many nation-states in Europe resorted to forced assimilation, oppression of minorities and sometimes even to population transfers and ethnic cleansing in order to create the homogeneous nation that the model seemed to require. Brubaker even argues that trans-border migration, which has always been a quite normal phenomenon in human history, in fact contradicts the nation-state model, since it both undermines the cultural unity of the nation and the sharp borders between the supposedly unique national cultures. Berger and Miller add that nation-states and empires were not mutually exclusive and that actually most European nation-states during the nineteenth and twentieth centuries were ‘nationalizing empires’. It can be assumed that outside of Europe the boundaries between both were not that sharp either. Even in the colonies nationalizing tendencies were detectable, for instance when mass media began to construct a collective ‘national’ identity for each colony, even long before the first movements of national liberation came into being.

In light of the very brief period in which the nation-state model was truly hegemonic, it is surprising that nationalism had such a profound and lasting impact on the social sciences and the discipline of history. From the early nineteenth century onwards the nation-state became the dominant unit of analysis, which is still very much the case; and, as Berger and Wimmer and Glick Schiller have made clear, it will not be an easy task to overcome the existing methodological nationalism. First of all, education and research is still organized along national lines. National archives collect primary sources along national lines, while national libraries do the same for secondary studies. Universities depend largely on the government and national agencies for their money and research funds. Many chairs in history departments are still dedicated to national history, while many conferences, associations and journals still focus on one national context. Maps and catalogues reproduce a world of bounded nation-states, while history books in libraries and shops are generally ordered according to the nationality of the topic.

It must be admitted that recently historians (and other scholars within the humanities and social sciences) have become more critical about the dominant position of the nation-state and have adapted their research agenda by, for instance, paying more attention to comparative history, transnational topics, or by exploring local, regional, imperial or global topics. Nevertheless, most scholars continue to focus on the local, regional or imperial past of one nation-state, take nation-states as their basic units for comparison or study the movement of persons, ideas or goods over *national* borders.

Historical handbooks – and this is even more the case with books that target a broad audience – still routinely use nation-states as the main territorial unit to structure their story and even project nations back into the distant past. Even the terminology we use is profoundly affected by historiographical nationalism. Thus, terms used for historical epochs, such as the Victorian era, *Vormärz*, the Third Republic, the Antebellum period and the *Risorgimento* are often derived from and applicable to one national context only. Moreover, supposed turning points are different for each national case, which makes comparison even more difficult. This is even more evident in the use of concepts that only apply to one national context, such as frontier, Home Rule, *Heimat*, *laicité* or *kulak*. Sometimes, even comparable political currents or movements receive different labels in each national context. Thus, German *Sozial-Liberalismus* is known as New Liberalism in Britain, *solidarisme* in France, *regeneracionismo* in Spain, the Giolittian Era in Italy and the Progressive Movement in the United States.

However, it is not just a question of units of analysis and terminology. The nation-state also provides a normative framework, which is still omnipresent in the writings of historians and passes largely unacknowledged. It is understandable that as private individuals we prefer to live in a (democratic) nation-state, however, as professional historians we should not privilege one state form over another and treat them on an equal basis and with a neutral tone. Nonetheless, this is generally not the case. Like Wilson, historians still routinely describe empires as ‘backward’, while their policies are characterized as ‘foreign conquest’, ‘occupation’, ‘oppression’ and ‘subordination’, while the creation of nation-states is analysed by using positive terms like ‘revolution’, ‘unification’, ‘war of independence’, ‘liberation’ or ‘uprising’ (instead of, for instance, rebellion, secession, disruption or usurpation). Empires are described as ‘multi-ethnic’ or ‘multi-national’, thus implying that their demise was inevitable. Nation-states, on the other hand, are associated with progress, equality and self-determination, and cases that do not entirely conform to the ideal type are characterized as anomalies, such as the presence of ethnic minorities or immigrants, which have to be dealt with through nation-building or assimilation policies. Even postmodern and postcolonial concepts, such as ‘cross-cultural’, ‘othering’ or ‘hybrid identities’, presuppose that an independent, unified and homogenous nation-state is the norm. Thus, it will not be an easy task to overcome the methodological, terminological and normative nationalism that still dominates history writing. In order to succeed, we will have to amend our way of doing research, revise our vocabulary and rewrite the story of the past in a very thorough way.

## Notes

1 John Breuilly, ed., *The Oxford Handbook of the History of Nationalism* (Oxford 2013). The paperback edition appeared in the summer of 2016, which is fortunate since it could very well serve as an up-to-date handbook for undergraduate students.

2 See for instance Anthony D. Smith, *The Ethnic Origins of Nations* (Oxford 1986).

3 Azar Gat with Alexander Yakobson, *Nations: The Long History and Deep Roots of Political Ethnicity and Nationalism* (Cambridge 2013).

4 Azar Gat, *War in Human Societies* (Oxford 2008).

5 See for instance John Hutchinson, Chris Wickham, Bo Stråth and Azar Gat, ‘Debate on Azar Gat’s Nations: The Long History and Deep Roots of Political Ethnicity and Nationalism’, *Nations and Nationalism*, Vol. 21, No. 3 (2015), 383–402.

6 See for some innovative interpretations, based on more detailed case studies, that focus on continuity: Caspar Hirshi, *The Origins of Nationalism: An Alternative History from Ancient Rome to Early Modern Germany* (Cambridge 2011) and Lotte Jensen, ed., *The Roots of Nationalism: National Identity Formation in Early Modern Europe*, 1600–1815 (Amsterdam 2016).

7 Siniša Malešević, *Nation-States and Nationalisms: Organization, Ideology and Solidarity* (Cambridge 2013).

8 Charles S. Maier, *Once within Borders: Territories of Power, Wealth and Belonging Since 1500* (Cambridge 2016).

9 Andreas Wimmer, *Waves of War: Nationalism, State Formation and Ethnic Exclusion in the Modern World* (Cambridge 2013) 1–73.

10 Wimmer, *Waves of War*, 73–108.

11 Miguel Cabo and Fernando Molina, ‘The Long and Winding Road of Nationalization: Eugen Weber’s Peasants into Frenchmen in Modern European History (1976–2006)’, *European History Quarterly*, Vol. 39, No. 2 (2009), 264–86.

12 Michael Billig, *Banal Nationalism* (London 1995).

13 Tim Edensor, *National Identity, Popular Culture and Everyday Life* (Oxford 2002).

14 Atsuko Ichijo and Ronald Ranta, *Food, National Identity and Nationalism: From Everyday to Global Politics* (Basingstoke 2016).

15 Malešević, *Nation-States and Nationalisms*, 120–55.

16 Kolleen M. Guy, *When Champagne Became French: Wine and the Making of a National Identity* (Baltimore 2003); Steven L. Harp, *Marketing Michelin: Advertising and Cultural Identity in Twentieth-Century France* (Baltimore, MD 2001); Eric G. E. Zuelow, *Making Ireland Irish: Tourism and National Identity Since the Irish Civil War* (Syracuse, NY 2009).

17 Andreas Wimmer and Nina Glick Schiller, ‘Methodological Nationalism and Beyond: Nation-State Building, Migration and the Social Sciences’, *Global Networks*, Vol. 2, No. 4 (2002), 301–34; Daniel Chernillo, ‘The Critique of Methodological Nationalism: Theory and History’, *Thesis Eleven*, Vol. 106, No. 1 (2011), 98–117.

18 Stefan Berger with Christoph Conrad, *The Past as History: National Identity and Historical Consciousness in Modern Europe* (Basingstoke 2015). The author primarily concentrates on German, French and British historians, but, without being exhaustive, in almost all chapters of the book examples are given for a number of other countries, while highlighting differences and similarities between the various parts of Europe. In the end, considerable attention is given to almost all of the smaller European countries from Albania to Iceland and from Belorussia to Portugal.

19 See also Geneviève Warland, ‘Wars of Religion in National History Writing at the Beginning of the Twentieth Century: P. J. Blok, Karl Lamprecht, Ernest Lavisse and Henri Pirenne’, in Stefan Berger and Chris Lorenz, eds, *Nationalizing the Past: Historians as Nation Builders in Modern Europe* (Basingstoke 2010), 107–28.

20 Both were published in 2000. See also Stefan Berger, ‘Rising Like a Phoenix … The Renaissance of National History Writing in Germany and Britain since the 1980s’ in Stefan Berger and Chris Lorenz, eds, *Nationalizing the Past: Historians as Nation Builders in Modern Europe* (Basingstoke 2010), 426–52.

21 Rogers Brubaker, *Grounds for Difference* (Cambridge 2015), 152. See, for a recent, but not always convincing, defense of the multiple modernity thesis: Atsuko Ichijo, *Nationalism and Multiple Modernities: Europe and Beyond* (Basingstoke 2013).

22 Brubaker, *Grounds for Difference*, 20.

23 Ibid., 132.

24 Ibid., 99 and 101.

25 Stefan Berger and Alexei Miller, eds, *Nationalizing Empires* (Budapest 2015).

26 See, for recent historiographical trends, also the chapters by Ther and Leonhard.

27 A similar approach can be found in Pieter Judson, *The Habsburg Empire: A New History* (Cambridge 2016) and the special issue edited by Siniša Malešević, ‘Empires and Nation-States: Beyond the Dichotomy’, *Thesis Eleven* (2017), 3–10.

28 Róisín Healy and Enrico Dal Lago, eds, *The Shadow of Colonialism on Europe’s Modern Past* (Basingstoke 2014).

29 See, for an attempt to historicize banal forms of nationalism: Eric Storm, ‘The Nationalisation of the Domestic Sphere’, *Nations and Nationalism*, Vol. 23, No. 1 (2017), 173–93.

